# Supplementary material for: Parkinson’s disease linked LRRK2 G2019S drives oxidative nuclear DNA damage and PARP1 hyperactive signaling
Source: bioRxiv. 2026 Mar 1:2026.02.27.708379. Preprint. [Version 1] doi: 10.64898/2026.02.27.708379 (PMC13014154; doi:10.64898/2026.02.27.708379)
Supplement: Supplement 1 [file NIHPP2026.02.27.708379v1-supplement-1.pdf]

bioRxiv preprint doi: https://doi.org/10.64898/2026.02.27.708379; this version posted March 1, 2026. The copyright holder for this preprint (which was not certified by peer review) is the author/funder, who has granted bioRxiv a license to display the preprint in perpetuity. It is made available under aCC-BY 4.0 International license.

SUPPLEMENTAL FIGURES

bioRxiv preprint doi: https://doi.org/10.64898/2026.02.27.708379; this version posted March 1, 2026. The copyright holder for this preprint (which was not certified by peer review) is the author/funder, who has granted bioRxiv a license to display the preprint in perpetuity. It is made available under aCC-BY 4.0 International license.

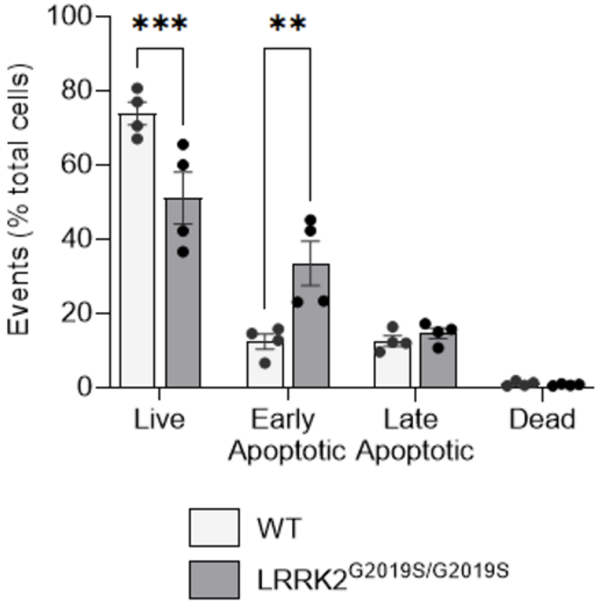

Supplementary Figure 1. LRRK2$^{G2019S/G2019S}$ cells have decreased viability compared to wild-type. Flow cytometry analysis of live, apoptotic, and necrotic cells in wild-type and LRRK2$^{G2019S/G2019S}$ cells. (n=4, **p < 0.01, ***p < 0.001 determined by two-way ANOVA with Bonferroni's multiple comparison). Data are presented as mean ± SEM.

bioRxiv preprint doi: https://doi.org/10.64898/2026.02.27.708379; this version posted March 1, 2026. The copyright holder for this preprint (which was not certified by peer review) is the author/funder, who has granted bioRxiv a license to display the preprint in perpetuity. It is made available under aCC-BY 4.0 International license.

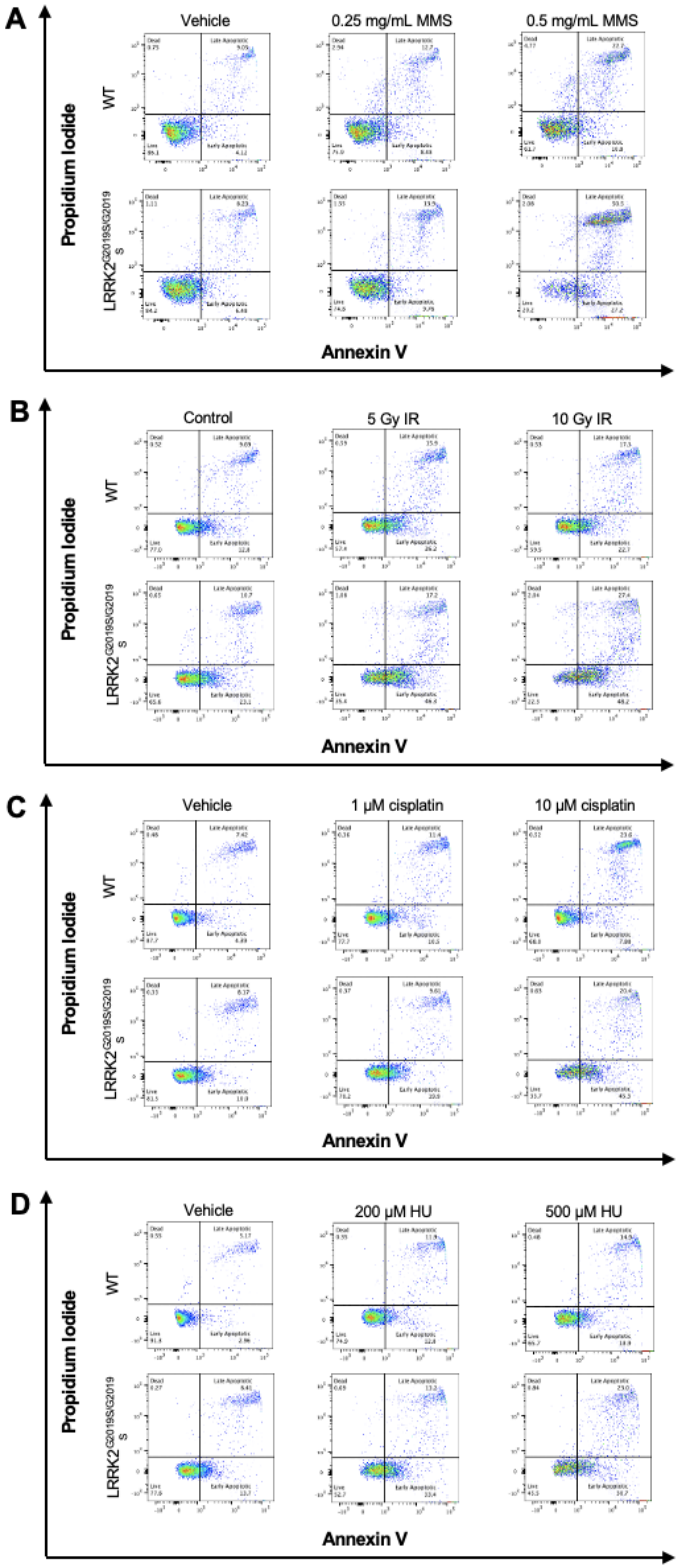

bioRxiv preprint doi: https://doi.org/10.64898/2026.02.27.708379; this version posted March 1, 2026. The copyright holder for this preprint (which was not certified by peer review) is the author/funder, who has granted bioRxiv a license to display the preprint in perpetuity. It is made available under aCC-BY 4.0 International license.

Supplementary Figure 2. The LRRK2 G2019S mutation sensitizes cells to DNA damaging agents *in vitro*. Representative flow cytometry plots of live, apoptotic, and necrotic cells in wild-type and LRRK2$^{G2019S/G2019S}$ KI cells exposed to (**A**) methyl methanesulfonate (MMS), (**B**) ionizing radiation (IR), (**C**) cisplatin, or (**D**) hydroxyurea (HU) and stained for annexin V/propidium iodide (PI).

bioRxiv preprint doi: https://doi.org/10.64898/2026.02.27.708379; this version posted March 1, 2026. The copyright holder for this preprint (which was not certified by peer review) is the author/funder, who has granted bioRxiv a license to display the preprint in perpetuity. It is made available under aCC-BY 4.0 International license.

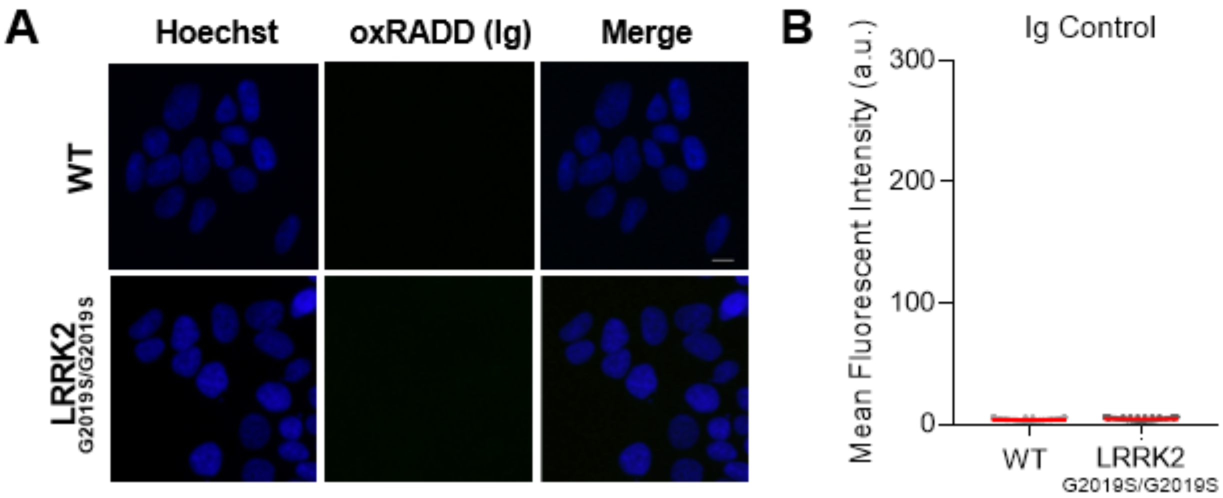

Supplementary Figure 3. Isotype controls in wild-type and LRRK2$^{G2019S/G2019S}$ cells for the oxRADD assay. (**A**) Representative 20X wide-field images of wild-type (WT) and LRRK2$^{G2019S/G2019S}$ KI cells incubated with Hoechst (blue) and isotype control (green). (**B**) Quantification of IgG control (n=3). Approximately 1700-2000 total cells were analyzed from three independent experiments. Data are presented as mean ± SEM.

bioRxiv preprint doi: https://doi.org/10.64898/2026.02.27.708379; this version posted March 1, 2026. The copyright holder for this preprint (which was not certified by peer review) is the author/funder, who has granted bioRxiv a license to display the preprint in perpetuity. It is made available under aCC-BY 4.0 International license.

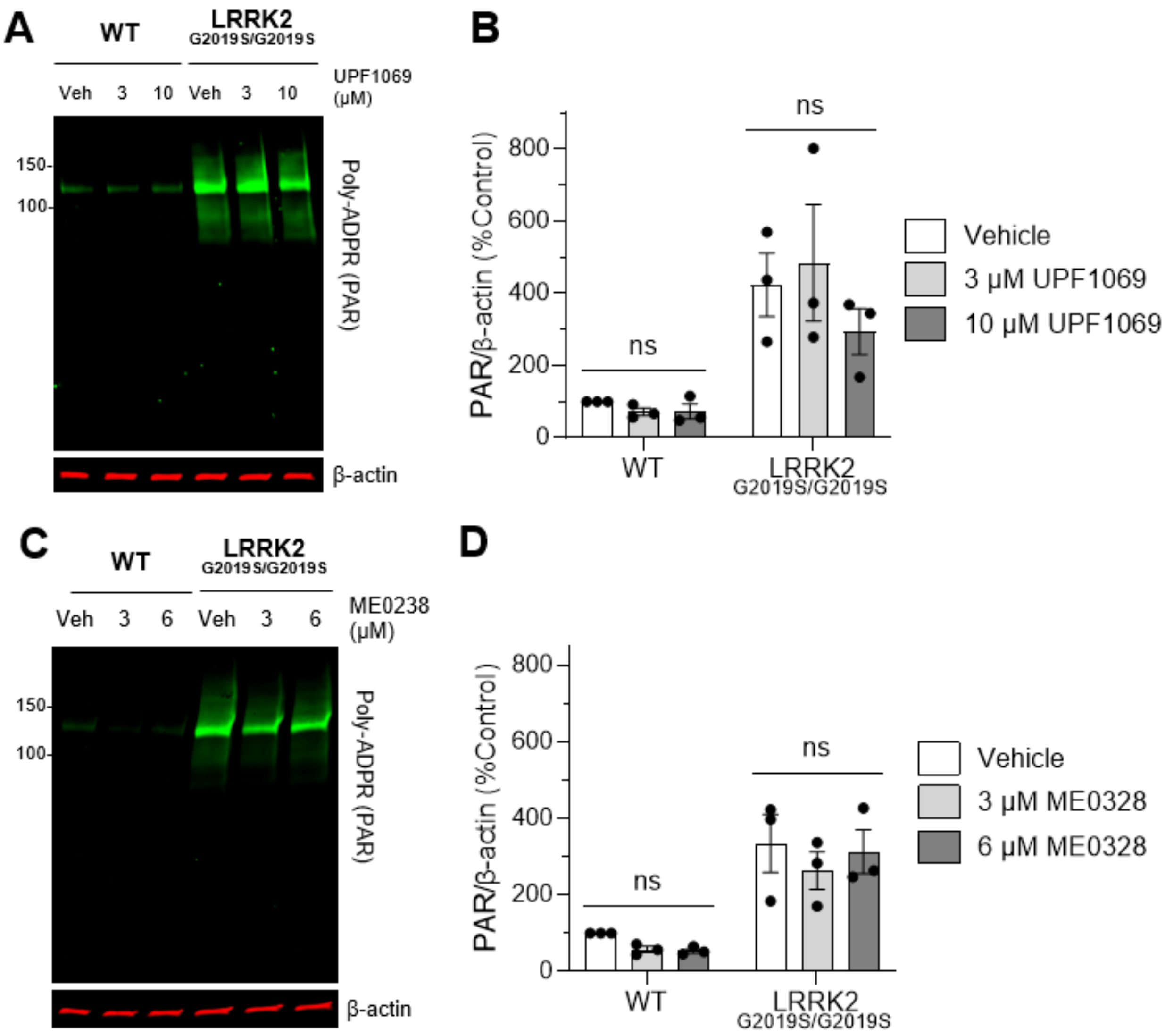

Supplementary Figure 4. LRRK2 G2019S-mediated PAR accumulation *in vitro* is not PARP2- or PARP3-dependent. (**A**) Representative western blot of wild-type and LRRK2$^{G2019S/G2019S}$ KI cells treated with vehicle or the PARP2-selective inhibitor UPF1069 (3 and 10 µM) and assessed for PAR and β-actin as a loading control. (**B**) No differences in quantification of PAR levels with treatment in either cell line. (n=3, ns, determined by two-way ANOVA with Bonferroni's multiple comparison). (**C**) Representative western blot of wild-type and LRRK2$^{G2019S/G2019S}$ KI cells treated vehicle or the PARP3-selective inhibitor ME0238 (3 and 6 µM) and assessed for PAR and β-actin as a loading control. (**D**) No differences in quantification of PAR levels with treatment in either cell line. (n=3, ns, determined by two-way ANOVA with Bonferroni's multiple comparison). Data are presented as mean ± SEM.

bioRxiv preprint doi: https://doi.org/10.64898/2026.02.27.708379; this version posted March 1, 2026. The copyright holder for this preprint (which was not certified by peer review) is the author/funder, who has granted bioRxiv a license to display the preprint in perpetuity. It is made available under aCC-BY 4.0 International license.

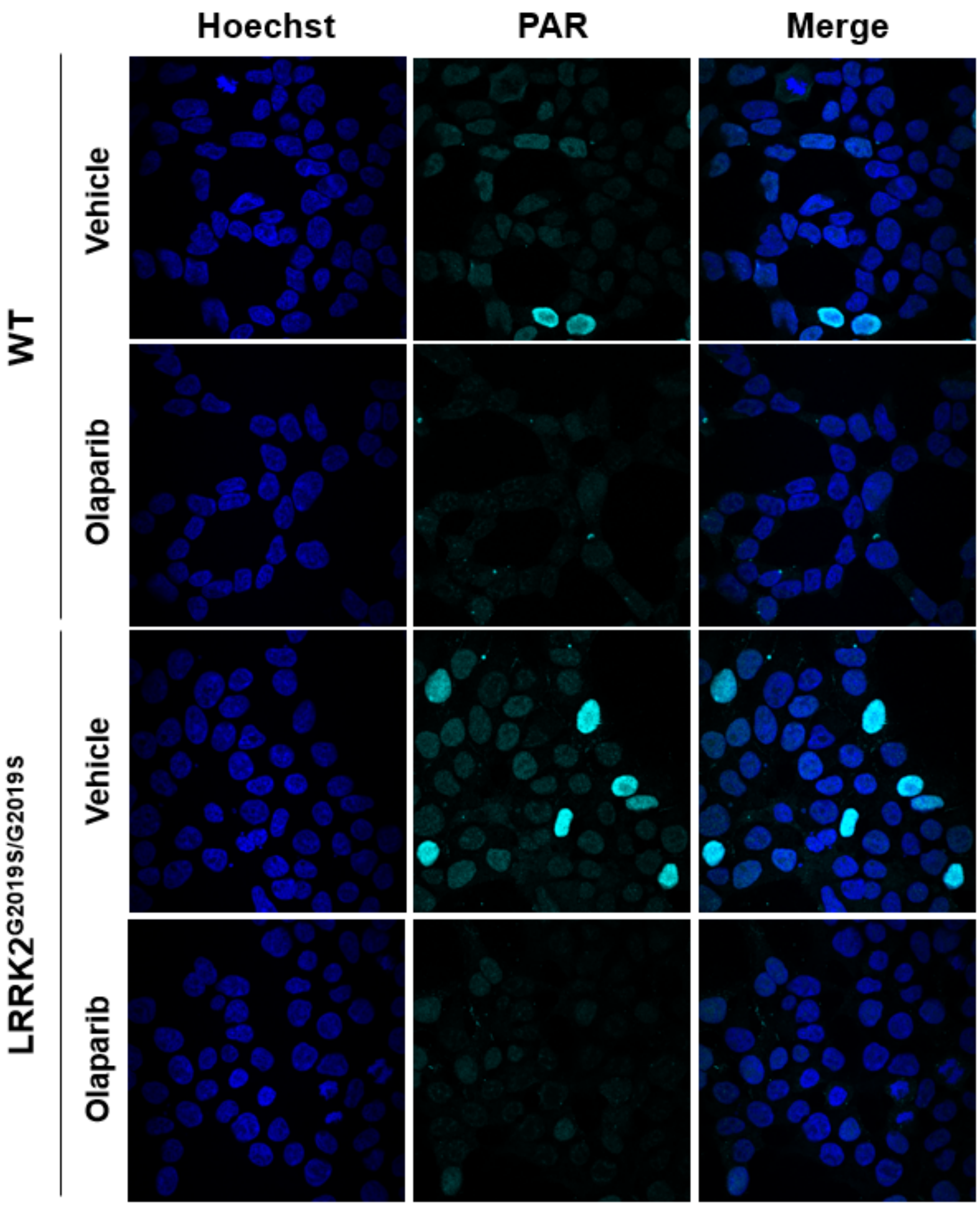

Supplementary Figure 5. PAR signal is abrogated with olaparib treatment in wild-type and LRRK2$^{G2019S/G2019S}$ cells. Representative 60X confocal fluorescence images of wild-type and LRRK2$^{G2019S/G2019S}$ cells incubated with vehicle or olaparib (10 μM) for 1 h.

bioRxiv preprint doi: https://doi.org/10.64898/2026.02.27.708379; this version posted March 1, 2026. The copyright holder for this preprint (which was not certified by peer review) is the author/funder, who has granted bioRxiv a license to display the preprint in perpetuity. It is made available under aCC-BY 4.0 International license.

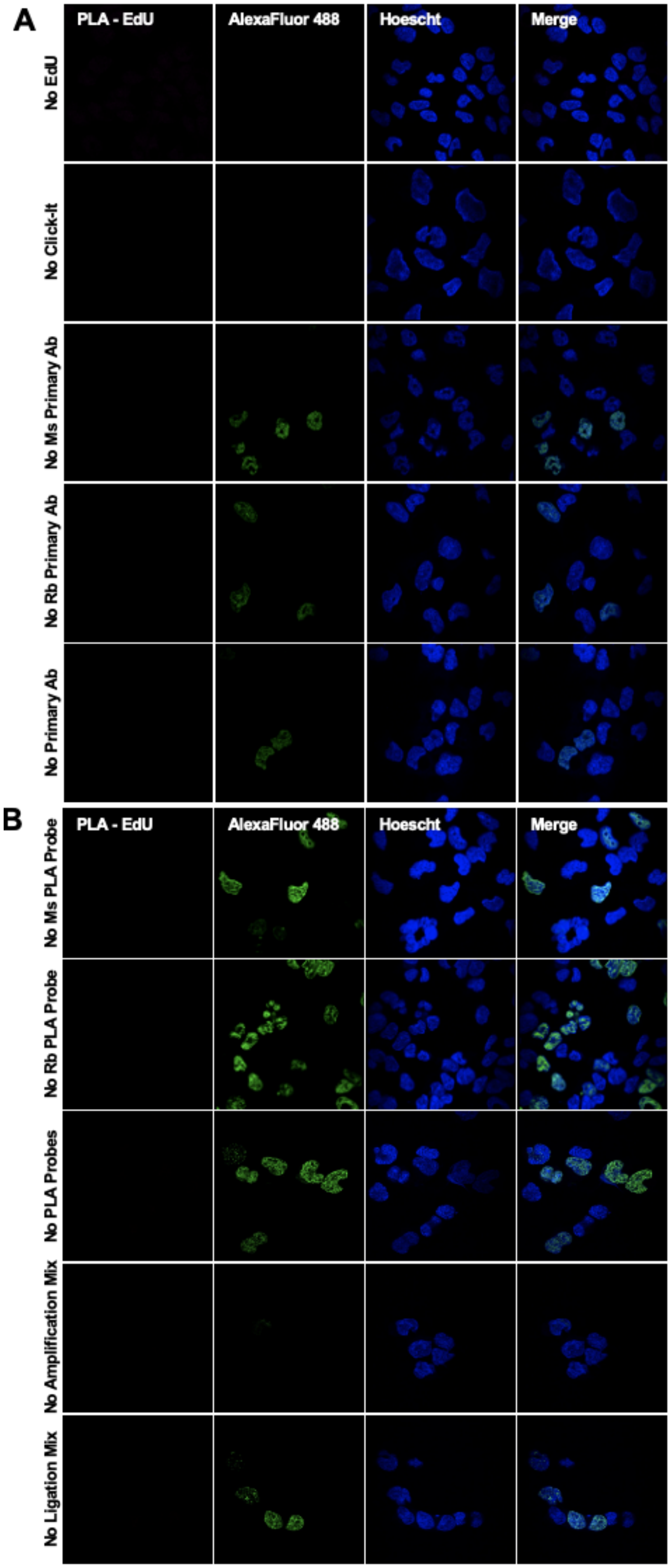

bioRxiv preprint doi: https://doi.org/10.64898/2026.02.27.708379; this version posted March 1, 2026. The copyright holder for this preprint (which was not certified by peer review) is the author/funder, who has granted bioRxiv a license to display the preprint in perpetuity. It is made available under aCC-BY 4.0 International license.

Supplementary Figure 6. Proximity ligation assay controls. To assess for non-specific or potential background staining in PLA staining, we performed the MIRA protocol in (**A**) WT-LRRK2 cells without the addition of EdU, omitting the steps of the Click-IT reaction, or no addition of the individual mouse or rabbit primary antibody, or primary antibodies, and additional controls that included (**B**) lack of individual mouse or rabbit PLA probes, or both PLA probes, or the PLA amplification or ligation mix was excluded.

bioRxiv preprint doi: https://doi.org/10.64898/2026.02.27.708379; this version posted March 1, 2026. The copyright holder for this preprint (which was not certified by peer review) is the author/funder, who has granted bioRxiv a license to display the preprint in perpetuity. It is made available under aCC-BY 4.0 International license.

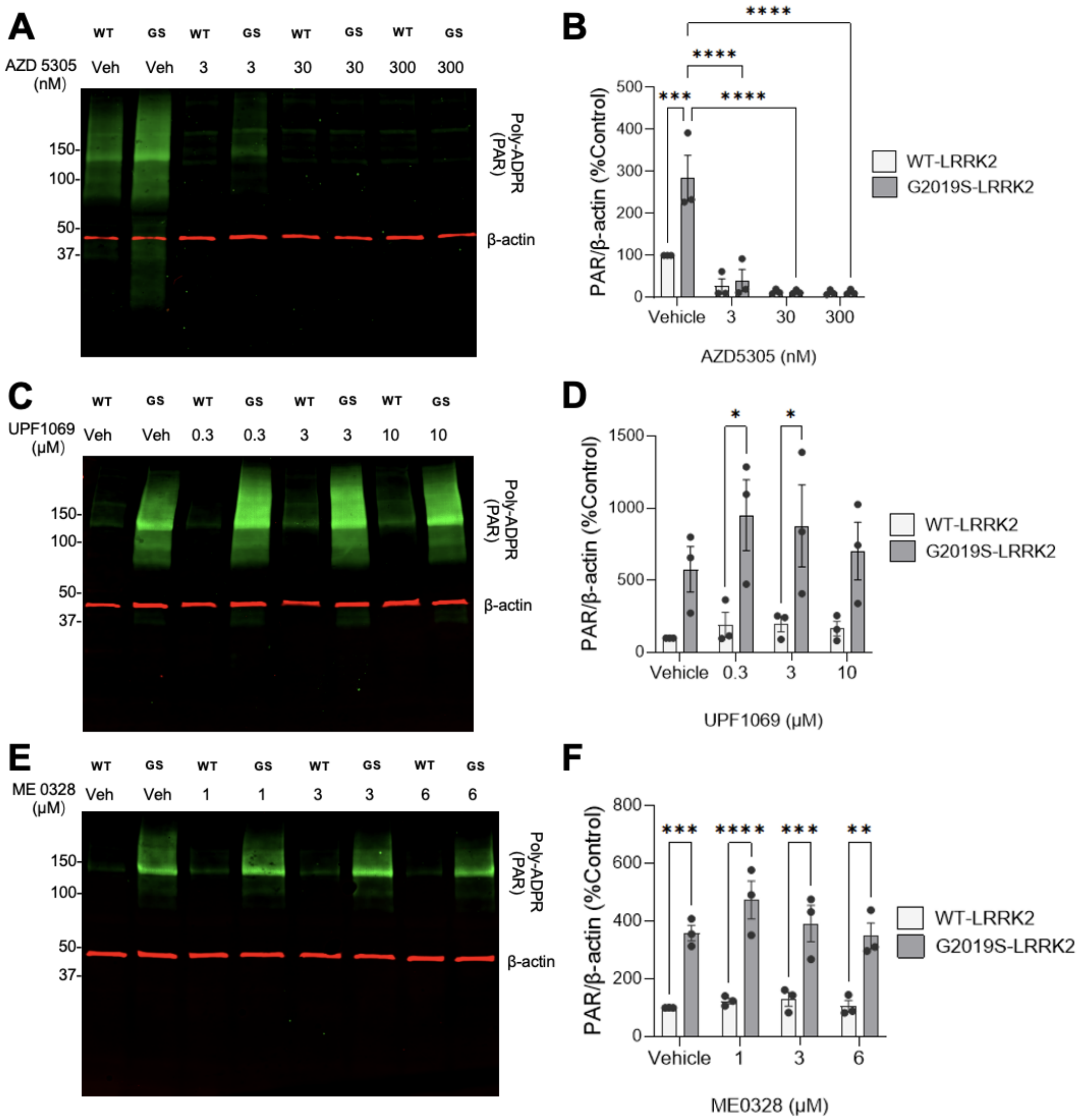

Supplementary Figure 7. G2019S-LRRK2 mediated PAR accumulation is PARP1-dependent *in vitro*. (**A**) Representative western blot of WT-LRRK2 and G2019S-LRRK2 cells treated with vehicle or the PARP1-selective inhibitor AZD5305 (3, 30 and 300 nM) and assessed for PAR and β-actin as a loading control. (**B**) Quantification demonstrated PAR levels are PARP1-dependent. (n =3, ***p < 0.001, ****p < 0.0001, determined by two-way ANOVA with Bonferroni's multiple comparison). (**C**) Representative western blot of WT-LRRK2 and G2019S-LRRK2 cells treated with vehicle or the PARP2-selective inhibitor UPF1069 (0.3, 3 and 10 μM) and assessed for PAR and β-actin as a loading control. (**D**) No differences in quantification of PAR levels with treatment in either cell line. (n=3, ns, determined by two-way ANOVA with Bonferroni's multiple comparison). (**E**) Representative western blot of WT-LRRK2 and G2019S-LRRK2 cells treated with vehicle or the PARP3-selective inhibitor ME0238 (1, 3 and 6 μM) and assessed for PAR and β-actin as a loading

bioRxiv preprint doi: https://doi.org/10.64898/2026.02.27.708379; this version posted March 1, 2026. The copyright holder for this preprint (which was not certified by peer review) is the author/funder, who has granted bioRxiv a license to display the preprint in perpetuity. It is made available under aCC-BY 4.0 International license.

control. (**F**) No differences in quantification of PAR levels with treatment in either cell line. (n=3, ns, determined by two-way ANOVA with Bonferroni's multiple comparison). Data are presented as mean ± SEM.

bioRxiv preprint doi: https://doi.org/10.64898/2026.02.27.708379; this version posted March 1, 2026. The copyright holder for this preprint (which was not certified by peer review) is the author/funder, who has granted bioRxiv a license to display the preprint in perpetuity. It is made available under aCC-BY 4.0 International license.

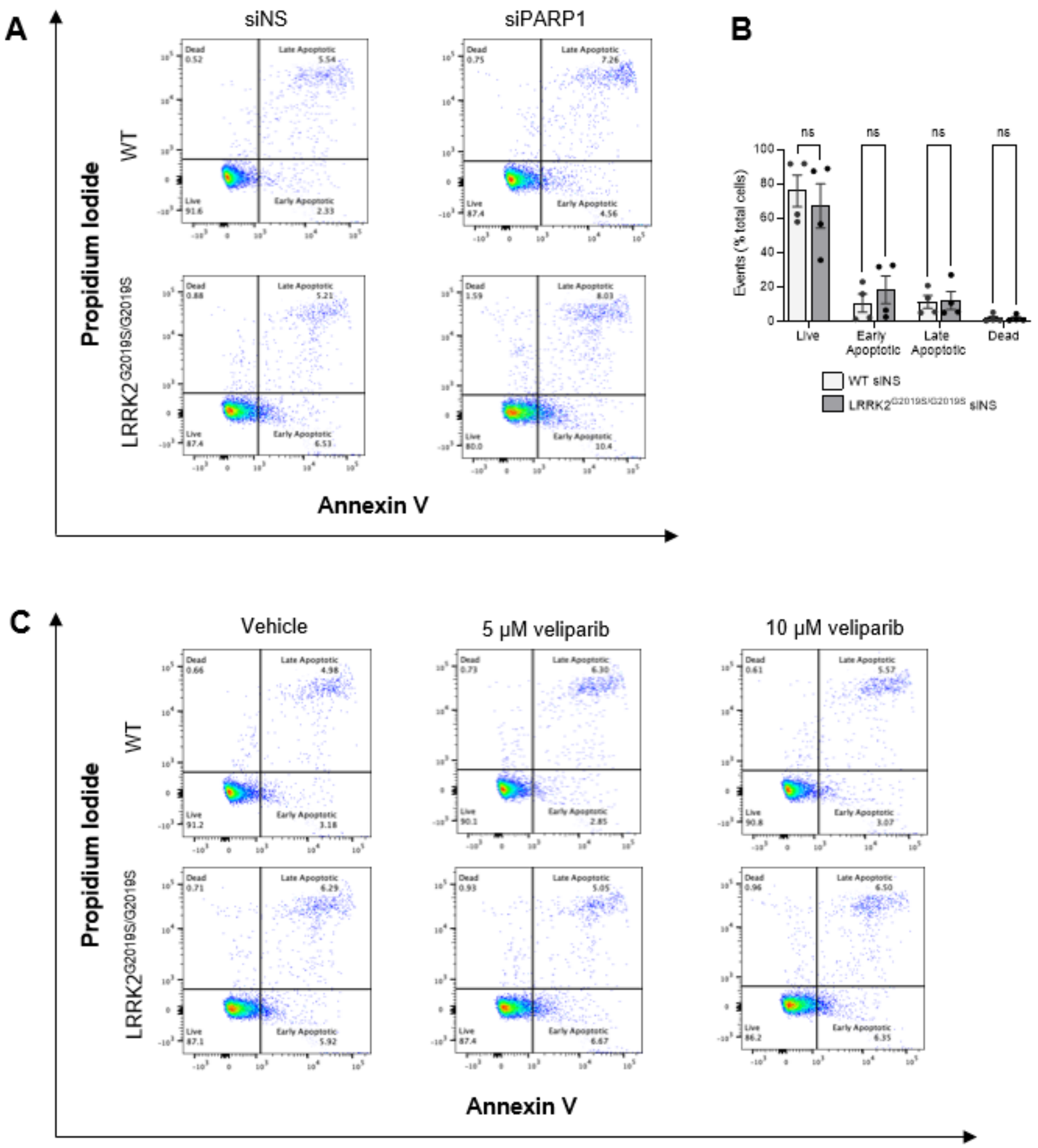

Supplementary Figure 8. Viability of wild-type and LRRK2$^{G2019S/S2019S}$ cells are similar following either PARP1 knock-down or inhibition with veliparib. (**A**) Representative flow cytometry plots of wild-type and LRRK2$^{G2019S/G2019S}$ KI cells stained with annexin V/propidium iodide (PI) after transient knock-down with siNS or siPARP1 (48 h). (**B**) Live, apoptotic, and necrotic populations of wild-type and LRRK2$^{G2019S/G2019S}$ KI cells after transfection with scramble siRNA (siNS). (n=4, ns = non-significant, determined by two-way ANOVA with Bonferroni's multiple comparison). (**C**) Representative flow cytometry plots of wild-type and LRRK2$^{G2019S/G2019S}$ KI cells treated with vehicle or veliparib and stained with annexin V/PI. Data are presented as mean ± SEM.

bioRxiv preprint doi: https://doi.org/10.64898/2026.02.27.708379; this version posted March 1, 2026. The copyright holder for this preprint (which was not certified by peer review) is the author/funder, who has granted bioRxiv a license to display the preprint in perpetuity. It is made available under aCC-BY 4.0 International license.

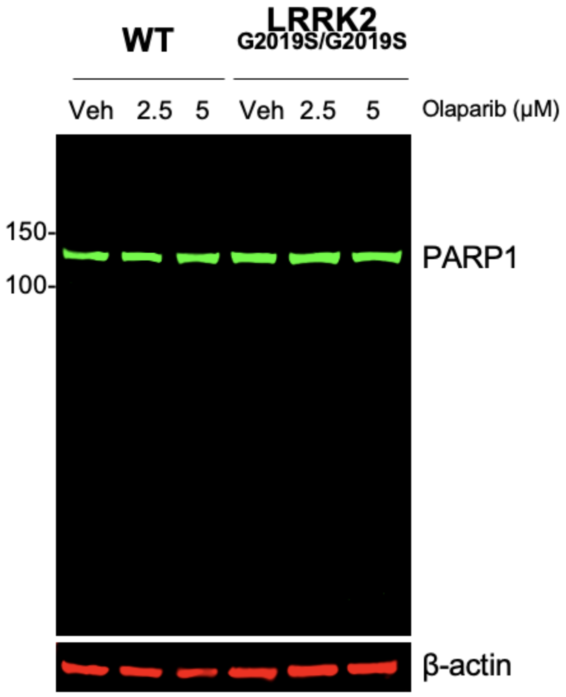

Supplementary Figure 9. LRRK2$^{G2019S/G2019S}$ KI and wild-type cells do not exhibit PARP1 cleavage after treatment with olaparib. Representative western blot of wild-type (WT) and LRRK2$^{G2019S/G2019S}$ KI cells treated with vehicle or olaparib (2.5 and 5 µM) for 48 h and assessed for PARP1 and β-actin as loading control.

bioRxiv preprint doi: https://doi.org/10.64898/2026.02.27.708379; this version posted March 1, 2026. The copyright holder for this preprint (which was not certified by peer review) is the author/funder, who has granted bioRxiv a license to display the preprint in perpetuity. It is made available under aCC-BY 4.0 International license.

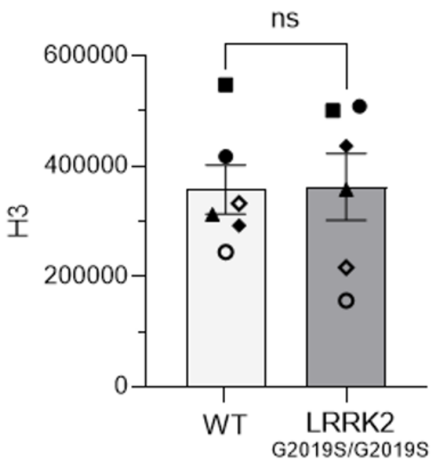

Supplementary Figure 10. H3 levels are equivalent in chromatin-enriched fractions between LRRK2$^{G2019S/S2019S}$ KI and wild-type cells. Quantification of H3 levels in chromatin-enriched fractions from wild-type (WT) and LRRK2$^{G2019S/G2019S}$ KI cells. (n=6, ns, determined by an unpaired t-test). Data are presented as mean ± SEM.

bioRxiv preprint doi: https://doi.org/10.64898/2026.02.27.708379; this version posted March 1, 2026. The copyright holder for this preprint (which was not certified by peer review) is the author/funder, who has granted bioRxiv a license to display the preprint in perpetuity. It is made available under aCC-BY 4.0 International license.

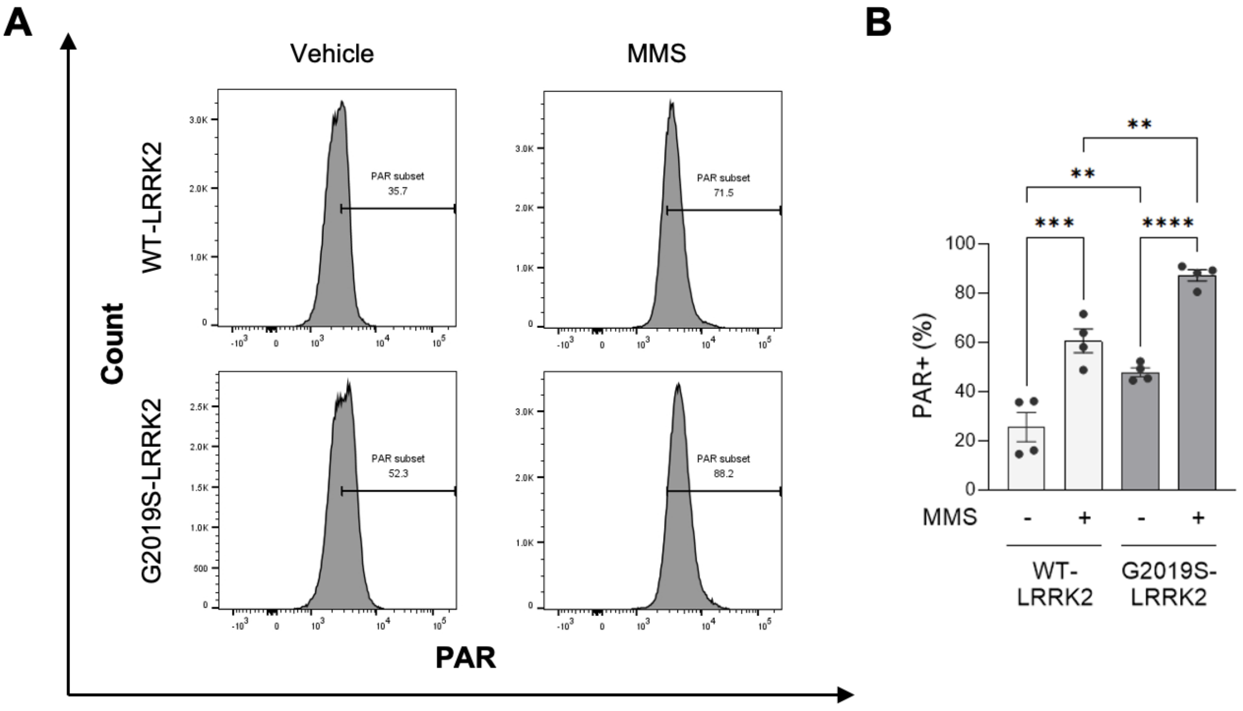

Supplementary Figure 11. The LRRK2 G2019S mutation sensitizes cells to the alkylating DNA damaging agent *in vitro*. (**A**) Representative flow cytometry plots of WT-LRRK2 and G2019S-LRRK2 expressing cells treated with vehicle or methyl methanesulfonate (MMS, 0.5 μM for 30 min). (**B**) Quantification of PAR-positive cells with and without exposure to MMS. (n=4, **p < 0.01, ***p < 0.001, ****p < 0.0001, determined by one-way ANOVA.) Data are presented as mean ± SEM.

bioRxiv preprint doi: https://doi.org/10.64898/2026.02.27.708379; this version posted March 1, 2026. The copyright holder for this preprint (which was not certified by peer review) is the author/funder, who has granted bioRxiv a license to display the preprint in perpetuity. It is made available under aCC-BY 4.0 International license.

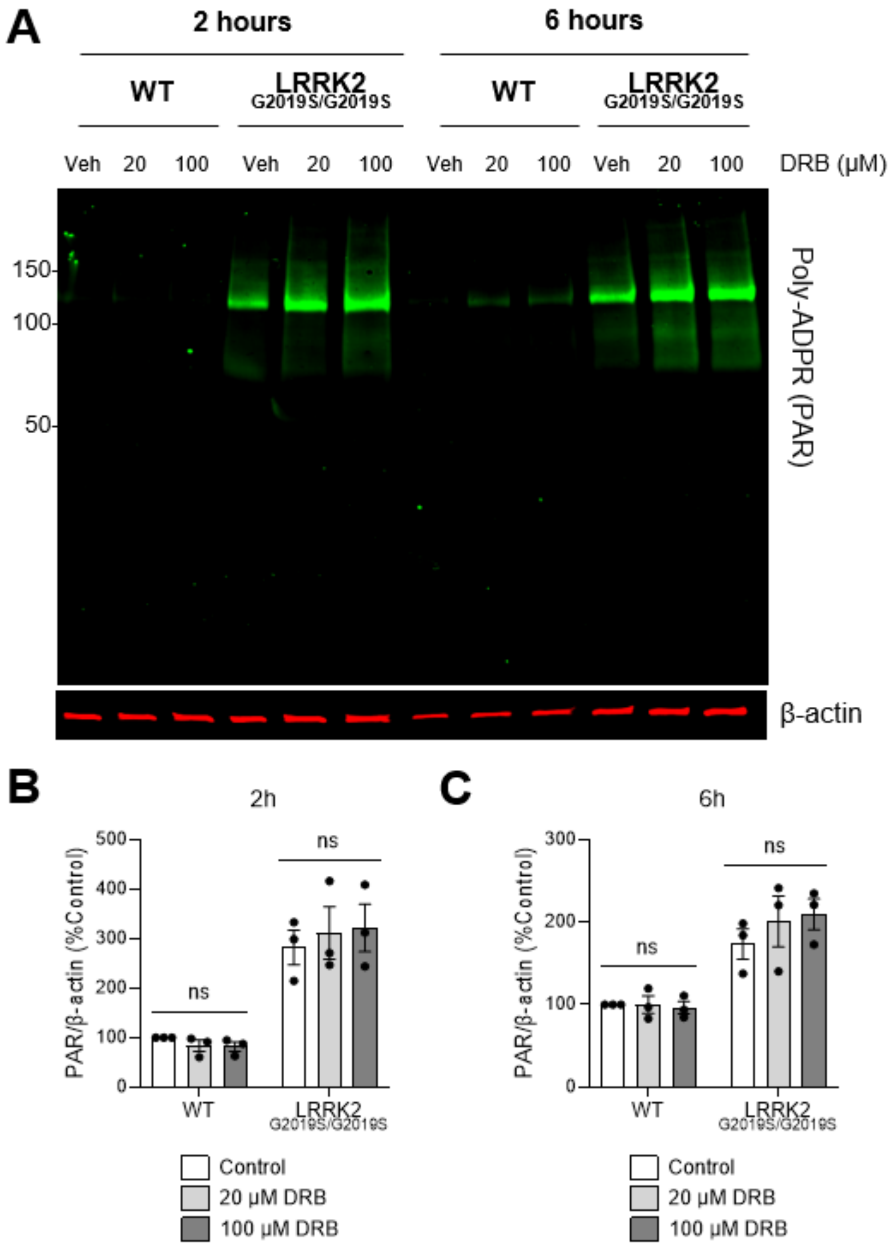

Supplementary Figure 12. LRRK2 G2019S-mediated PAR accumulation *in vitro* is not driven by transcription-related processes. (**A**) Representative western blots of wild-type (WT) and LRRK2$^{G2019S/G2019S}$ KI cells after treatment with vehicle or RNA Polymerase II inhibition with 5,6-dichloro-1-beta-D-ribofuranosylbenzimidazole (DRB) (20 and 100 μM) for 2 or 6 h and assessed for PAR levels and β-actin as a loading control. (**B**) Quantification of PAR levels were unchanged independent of genotype with either a 2 h treatment or (**C**) 6 h treatment. (n=3, ns, determined by two-way ANOVA with Bonferroni's multiple comparison). Data are presented as mean ± SEM.
